# New Estimators of the Bayes Factor for Models with High-Dimensional Parameter and/or Latent Variable Spaces

**DOI:** 10.3390/e23040399

**Published:** 2021-03-27

**Authors:** Anna Pajor

**Affiliations:** 1 Department of Mathematics, Cracow University of Economics, ul. Rakowicka 27, 31-510 Kraków, Poland; pajora@uek.krakow.pl or anna.1.pajor@uj.edu.pl; 2 Department of Financial Mathematics, Jagiellonian University in Kraków, ul. Prof. Stanisława Łojasiewicza 6, 30-348 Kraków, Poland

**Keywords:** Bayesian model comparison, Markov chain Monte Carlo, stochastic volatility

## Abstract

Formal Bayesian comparison of two competing models, based on the posterior odds ratio, amounts to estimation of the Bayes factor, which is equal to the ratio of respective two marginal data density values. In models with a large number of parameters and/or latent variables, they are expressed by high-dimensional integrals, which are often computationally infeasible. Therefore, other methods of evaluation of the Bayes factor are needed. In this paper, a new method of estimation of the Bayes factor is proposed. Simulation examples confirm good performance of the proposed estimators. Finally, these new estimators are used to formally compare different hybrid Multivariate Stochastic Volatility–Multivariate Generalized Autoregressive Conditional Heteroskedasticity (MSV-MGARCH) models which have a large number of latent variables. The empirical results show, among other things, that the validity of reduction of the hybrid MSV-MGARCH model to the MGARCH specification depends on the analyzed data set as well as on prior assumptions about model parameters.

## 1. Introduction

The Bayes factor, defined as a ratio of the marginal likelihoods for the two models being compared, is an important quantity in Bayesian model comparison and hypothesis testing, see, e.g., in [1]. The posterior odds ratio, used for comparing two competing models, is equal to the product of the prior odds and the Bayes’ factor. Therefore, one of the challenges for Bayesians is to accurately estimate the factor, especially in models with a large number of parameters and/or latent variables. Most popular methods of calculating the Bayes factor, based on estimation of the marginal likelihoods of each model separately, are very time-consuming (or even computationally infeasible) in high-dimensional cases. Therefore, methods for direct estimation of the Bayes factor, instead of estimating two marginal likelihoods, have been devised. There already exist different approaches, such as the product-space approach, reversible jump MCMC, and path sampling (see, e.g., in [2,3,4,5]), which make it possible to estimate the Bayes factor without calculation of the marginal densities of the data, but in many empirical cases (especially in the case of a large number of model parameters) they require extremely complicated and extensively time-consuming operations, with no general guarantees of success.

Note that for nested models with a large number of latent variables such as stochastic volatility models, Jacquier et al. [6] have shown how to estimate the Bayes factor directly using Markov Chain Monte Carlo (MCMC) simulations from the posterior distribution. They pointed out that the Bayes factor can be expressed as the expected value of the ratio of corresponding densities with respect to the posterior distribution of parameters and latent variables. This expected value can be estimated by averaging over the MCMC draws. Thus, using the special structure of stochastic volatility models and exploiting prior assumptions about parameters, they have estimated Bayes factors for comparing the basic stochastic volatility model to the models with fat tails and correlated errors. Unfortunately, the finite sample properties of the estimator have not been analyzed by the authors. In this paper, we will show that even in very simple models it does not perform well, but it can be improved by trimming the space of parameters and latent variables.

Now, the issue of a direct estimation of the Bayes factor will be described more formally. Let us consider a Bayesian model, in which

(1) the space of parameters is denoted by Θ and p(θ) is a prior density function of the parameters collected in θ∈Θ,
(2) the space of latent variables is denoted by *H* and p(h|θ) is a prior density function of the latent variables collected in h∈H, and
(3) *y* is a vector of observations.

The marginal data density, p(y), is defined as the integral (calculated over the whole space of parameters and latent variables, Θ×H) of the conditional data density given the vector of parameters and latent variables, p(y|θ,h), with respect to the prior distribution of the parameters and latent variables:

(1) $$p\left(y\right)=\int_{\Theta \times H}p(y,h,\theta )\;d\theta \;dh=\int_{\Theta \times H}p(y|h,\theta )\;p(h|\theta )\;p(\theta )\;d\theta \;dh.$$

In the majority of models it is impossible to analytically integrate out parameters and latent variables from the joint distribution for *y*, *h*, and θ, p(y,h,θ). It very often results from the number of dimensions of the space of the latent variables and parameters being too large. Furthermore, a correct assessment of the marginal likelihood is computationally challenging (see, e.g., in [7,8,9], where summaries of various methods can be found). In the presence of a large number of latent variables (e.g., in stochastic volatility models) popular methods of Monte Carlo estimation of p(y) for the observed vector *y* are computationally extremely intensive, and often they turn out to be infeasible. Fortunately, we can consider the ratio of marginal data densities instead of p(y). In fact, the main criterion of comparison of two competing models $M_{i}$ and $M_{j}$ is the posterior odds ratio between the two models:

(2) $$O_{ij}=\frac{p(M_{i}|y)}{p(M_{j}|y)}=\frac{p(y|M_{i})}{p(y|M_{j})}\times \frac{p(M_{i})}{p(M_{j})},$$

where $p(M_{i}|y)$ is the posterior probability of the model $M_{i}$, $p(M_{i})$ is the prior probability of the model $M_{i}$, and $p\left(y|M_{i}\right)=\int_{\Theta_{i}\times H_{i}}p\left(y|h_{i},\theta_{i},M_{i}\right)\;p\left(h_{i}|\theta_{i},M_{i}\right)\;p\left(\theta_{i}|M_{i}\right)\;d\theta_{i}\;dh_{i}$ is the marginal data density value (the marginal likelihood) in the model $M_{i}.$ The ratio $B_{ij}=\frac{p(y|M_{i})}{p(y|M_{j})}$ is referred to as the Bayes factor in favor of the model $M_{i}$ against the model $M_{j}$, in turn, the ratio $\frac{p(M_{i})}{p(M_{j})}$ is the prior odds ratio. Thus, if the prior odds ratio is equal to 1 (i.e., both models are equally probable a priori, $p\left(M_{i}\right)=p\left(M_{j}\right))$, the posterior odds ratio between the two models is then equal to the Bayes factor. Moreover, if we assume that the set of models $\left\{M_{1},\ldots ,M_{m}\right\}$ is exhaustive, Bayes factors can be used to obtain the posterior model probabilities:

(3) $$p\left(M_{i}|y\right)=\frac{\frac{p\left(M_{i}\right)p\left(y|M_{i}\right)}{p(y|M_{k})}}{\frac{\sum_{j=1}^{m}p\left(M_{j}\right)p\left(y|M_{j}\right)}{p(y|M_{k})}}=\frac{p\left(M_{i}\right)B_{ik}}{\sum_{j=1}^{m}p\left(M_{j}\right)B_{jk}},$$

for i,k∈{1,⋯,m}. By choosing k=i, we obtain

(4) $$p\left(M_{i}|y\right)=\frac{p(M_{i})}{\sum_{j=1}^{m}p\left(M_{j}\right)B_{ji}},$$

for i∈{1,⋯,m}.

Thus, the Bayes factors can be used instead of the marginal data density values. Note that in many situations it is easier to estimate the Bayes factor than the marginal likelihood. In this paper, we show how to estimate the Bayes factor in models with a large number of parameters and/or latent variables, in which calculation of the marginal data density value is numerically impossible. It will be shown that the Bayes factor is equal to the posterior mean, restricted to a certain subset *D* of the parameter and latent variable space, of the ratio of conditional densities of the corresponding quantities times the reciprocal of the posterior probability of the subset *D*. This fact leads the researcher to using arithmetic mean estimator of the ratio based on simulation from the posterior distribution restricted to the subset *D*.

It is well known that the Savage–Dickey density ratio and its generalizations (see in [10,11,12]) are relatively simple and widely used methods for computing Bayes factors for nested models. The Savage–Dickey method requires an estimate of the value of the posterior distribution at a single point. It is not reliable when this point lies in the tail of the posterior distribution. As has already been mentioned, the Savage–Dickey method can be used only for nested models. Our method can be applied for both nested and non-nested ones.

Note that although the posterior odds principle is fundamental, there are other Bayesian approaches to model comparison or hypothesis testing, e.g., the so-called Lindley type test (see [13]) or the Full Bayesian Significance Test (FBST), introduced by Pereira and Stern [14] as a Bayesian significance test for precise (sharp) hypotheses. A solid theoretical background of the FBST can be found in [15]. Detailed discussions and extensions of the Pereira–Stern test as well as of its applications are presented in, e.g., [16,17,18,19,20,21]. Our approach to Bayesian model comparison (or comparing hypotheses) is based on posterior probabilities associated with models (or hypotheses). This leads directly to the so-called posterior odds approach. Motivations for the formal Bayesian approach to model selection and for the use of Bayes factors are discussed in [22]. First of all, the Bayes factors and posterior model probabilities are easy to understand. Moreover, this approach is consistent, penalizes model complexity, remains conceptually the same regardless of the number of models (or hypotheses) under consideration, and does not require nested models or regular asymptotics. This approach allows one not only to test hypotheses, but also to compare them—“the process of revising prior probabilities associated with alternative hypotheses does not necessarily involve a decision to reject or accept these hypotheses” (see [1], p. 291). Furthermore, the Bayes factors make it possible to compare models whose prior distributions are different. Finally, the posterior probabilities of models or the Bayes factors can be used in the so-called Bayesian pooling approach (or Bayesian model averaging, see, e.g., in [23]). This paper is organized as follows. In Section 2, our new method is presented. Section 3 contains the simulation study. In Section 4, we present results of comparison of hybrid MSV-MGARCH models. The last section contains conclusions and direction of further research.

## 2. New Estimators of the Bayes Factor

It is easy to show that the Bayes factor in favor of the model $M_{i}$ against the model $M_{j}$ can be expressed as an integral:

(5) $$\frac{p(y|M_{i})}{p(y|M_{j})}=\int_{\Theta_{i}\times H_{i}}\frac{p\left(y|\theta {}_{i},h_{i},M_{i}\right)p\left(h_{i}|\theta {}_{i},M_{i}\right)p\left(\theta {}_{i}|M_{i}\right)\;}{p\left(y|\theta {}_{j},h_{j},M_{j}\right)p\left(h_{j}|\theta {}_{j},M_{j}\right)p\left(\theta {}_{j}|M_{j}\right)}p\left(\theta {}_{j},h_{j}|y,M_{j}\right)\;d\theta_{i}\;dh_{i},$$

where $p(y|\theta_{i},h_{i},M_{i})$, $p(h_{i}|\theta_{i},M_{i})$, and $p(\theta_{i}|M_{i})$ denote the conditional probability density function of the vector of observations (conditional sampling density), the conditional probability density function of latent variables, and the prior density of parameters in the model $M_{i}$, respectively. Note that when the two competing models $M_{i}$ and $M_{j}$ have the same parameter vector and the vector of latent variables (i.e., $\theta_{i}=\theta_{j}=\theta$, $h_{i}=h_{j}=h$, $\Theta_{i}=\Theta_{j}=\Theta$, $H_{i}=H_{j}=H)$, then the Bayes factor can be computed in a relatively straightforward way which does not require estimation of marginal data density values for each model separately. Of course, it is possible only if draws from one of the posterior distributions are available (see in [24]). We have

(6) $$BF_{ij}=\int_{\Theta \times H}r_{ij}\left(\theta ,h\right)\;p\left(\theta ,h|y,M_{j}\right)\;d\theta \;dh,$$

where

$$r_{ij}\left(\theta ,h\right)=\frac{p\left(y|\theta ,h,M_{i}\right)p\left(h|\theta ,M_{i}\right)p\left(\theta |M_{i}\right)\;}{p\left(y|\theta ,h,M_{j}\right)p\left(h|\theta ,M_{j}\right)p\left(\theta |M_{j}\right)}.$$

Therefore, given the sample ${\left\{\left(\theta^{\left(q\right)}{}^{\prime },h^{\left(q\right)}{}^{\prime }\right)\right\}}_{q=1}^{k}$ from the posterior distribution $p(\theta ,h|y,M_{j})$, an estimator of $BF_{ij}$ can be expressed as

(7) $${\hat{BF}}_{ij}=\frac{1}{k}\sum_{q=1}^{k}r_{ij}\left(\theta^{\left(q\right)},h^{\left(q\right)}\right).$$

As was pointed out in [24], it is crucial that the variability of the ratio $r_{ij}\left(\theta ,h\right)$ is small under the posterior distribution $p(\theta ,h|y,M_{j})$; otherwise, estimates of $BF_{ij}$ might be driven by few values of $h^{\left(q\right)}$ and $\theta^{\left(q\right)}$, and thus an extremely large simulation sample could be required to obtain adequate result. To deal with this problem, we propose a certain modification of the estimator (7). The idea of this modification is based on trimming the posterior sample to a certain subset of parameter and latent variable space, similarly to the idea of the correction of arithmetic mean estimator, proposed in [25]. Let us assume that D⊆Θ×H and $0<Pr(D|y,M_{i})<\infty$. The equality

(8) $$\frac{p(y|M_{i})}{p(y|M_{j})}\mathrm{Pr}\left(D|y,M_{i}\right)=\int_{\Theta \times H}I_{D}\left(\theta ,h\right)\frac{p(y|M_{i})}{p(y|M_{j})}\;p\left(\theta ,h|y,M_{i}\right)\;d\theta \;dh\;,$$

implies that

$$\frac{p(y|M_{i})}{p(y|M_{j})}\mathrm{Pr}\left(D|y,M_{i}\right)=\int_{\Theta \times H}I_{D}\left(\theta ,h\right)\frac{p(y,\theta ,h|M_{i})}{p(y|M_{j})}\;\;d\theta \;dh.$$

We can see immediately that

$$\frac{p(y|M_{i})}{p(y|M_{j})}\mathrm{Pr}\left(D|y,M_{i}\right)=\int_{\Theta \times H}I_{D}\left(\theta ,h\right)\frac{p\left(y|\theta ,h,M_{i}\right)p\left(\theta ,h|M_{i}\right)}{p\left(y|\theta ,h,M_{j}\right)p\left(\theta ,h|M_{j}\right)}\;p\left(\theta ,h|y,M_{j}\right)\;d\theta \;dh,$$

thus

(9) $$\frac{p(y|M_{i})}{p(y|M_{j})}=\frac{1}{\mathrm{Pr}(D|y,M_{i})}\int_{\Theta \times H}I_{D}\left(\theta ,h\right)r_{ij}\left(\theta ,h\right)\;p\left(\theta ,h|y,M_{j}\right)\;d\theta \;dh.$$

Equality (9) means that the Bayes factor can be expressed as a product of the reciprocal of the posterior probability of the subset *D* in the model $M_{i}$, $Pr(D|y,M_{i})$, and of the expected value of the indicator function of subset *D* times the ratio $r_{ij}\left(\theta ,h\right)$, $I_{D}\left(\theta ,h\right)r_{ij}\left(\theta ,h\right)$. This expected value is calculated with respect to the posterior distribution of the model parameters and latent variables in the model $M_{j}$. Therefore, given the sample ${\left\{\left(h^{\left(q\right)}{}^{\prime },\theta^{\left(q\right)}{}^{\prime }\right)\right\}}_{q=1}^{k}$ from the posterior distribution $p(\theta ,h|y,M_{j})$, an estimator of ${BF}_{ij}$ can be expressed as

(10) $${\hat{BF}}_{ij,D}=\frac{1}{\hat{P}r\left(D|y,M_{i}\right)}\left[\frac{1}{k}\sum_{q=1}^{k}I_{D}\left(\theta^{\left(q\right)},h^{\left(q\right)}\right)r_{ij}\left(\theta^{\left(q\right)},h^{\left(q\right)}\right)\right],$$

where $\hat{P}r\left(D|y,M_{i}\right)$ is an assessment of the posterior probability of the subset *D* in the model $M_{i}$. Unfortunately, this assessment requires also sampling from the posterior distribution in the model $M_{i}$.

Note that equality (9) can obviously be written as

(11) $$\frac{p(y|M_{i})}{p(y|M_{j})}=\frac{\mathrm{Pr}(D|y,M_{j})}{\mathrm{Pr}(D|y,M_{i})}\int_{D}r_{ij}\left(\theta ,h\right)\;p\left(\theta ,h|D,y,M_{j}\right)\;d\theta \;dh,$$

provided that $0<Pr(D|y,M_{j})<\infty$. This equality suggests that the Bayes factor can be estimated by the product of the ratio of posterior probabilities of the subset *D* and the sample arithmetic mean of the ratio $r_{ij}\left(\theta ,h\right)$:

(12) $${\hat{BF}}_{ij,D}=\frac{\hat{P}r\left(D|y,M_{j}\right)}{\hat{P}r\left(D|y,M_{i}\right)}\left[\frac{1}{k}\sum_{q=1}^{k}r_{ij}\left(\theta^{\left(q\right)},h^{\left(q\right)}\right)\right],$$

based on ${\left\{\left(h^{\left(q\right)}{}^{\prime },\theta^{\left(q\right)}{}^{\prime }\right)\right\}}_{q=1}^{k}$ drawn from the posterior distribution given by $p(\theta ,h|y,M_{j})$, truncated to the subset *D*, that is, $p(\theta ,h|D,y,M_{j})$.

As a by-product of our considerations, we have just shown that if a Monte Carlo sampler only visits a subset of the support of the posterior distribution, the correction of the sample arithmetic mean of the ratio $r_{ij}(\theta$,h) is needed.

Now, we will extend our analysis to models which contain two groups of parameters: the parameters common to both models and parameters specific to only one of them. Moreover, additional assumptions pertaining to conditional probability density functions will be discussed.

Let us assume that

(i) $\theta_{j}=\theta {}_{U}={\left(\theta_{A}^{\prime },\theta_{R}^{\prime }\right)}^{\prime }\in \Theta {}_{U}=\Theta {}_{A}\times \Theta_{R}$ denotes all the parameters of the model $M_{j}$ (since called $M_{U})$, while $\theta_{i}=\theta_{R}$ contains the parameters common to both models: $M_{U}$ and $M_{i}$ (since called $M_{R})$, and the vector $\theta_{A}$ denotes specific parameters of $M_{U}$;
(ii) $p\left(\theta_{U}|M_{U}\right)=p\left(\theta_{A},\theta_{R}|M_{U}\right)=p\left(\theta_{A}|M_{U}\right)p\left(\theta_{R}|M_{U}\right)$, i.e., the random vectors $\theta_{R}$ and $\theta_{A}$ are a priori independent;
(iii) $\;\int_{\Theta_{A}}p(\theta_{A}|M_{U})\;d\theta_{A}=1$, i.e., the prior distribution for the vector of parameters $\theta_{A}$ is proper; and
(iv) $h_{i}=h_{j}=h$; $H_{i}=H_{j}=H$, i.e., competing models have the same vector of latent variables.

### 2.1. Different Prior Distributions of Latent Variables in Both Models

In this section, we additionally assume that

(v) $p\left(y|\theta_{U},h_{U},M_{U}\right)=p\left(y|\theta_{R},h_{R},M_{R}\right)$, i.e., competing models have the same conditional sampling density and
(vi) $p\left(\theta_{R}|M_{U}\right)=p\left(\theta_{R}|M_{R}\right)$, i.e., in both models, the common parameters have the same prior distribution.

Under above assumptions the Bayes factor in favor of the model $M_{R}$ versus the model $M_{U}$ is given by the following integral:

(13) $$\frac{p(y|M_{R})}{p(y|M_{U})}=\int_{\Theta_{A}\times \Theta_{R}\times H}\frac{p(h|\theta_{R},M_{R})}{p(h|\theta_{A},\theta_{R},M_{U})}p\left(\theta_{A},\theta_{R},h|y,M_{U}\right)\;d\theta_{A}\;d\theta_{R}\;dh.$$

Thus, the estimator of the Bayes factor takes the form

(14) $${\hat{BF}}_{R,U,h}=\frac{1}{k}\sum_{q=1}^{k}\frac{p(h^{\left(q\right)}|\theta_{R}^{\left(q\right)},M_{R})}{p(h^{\left(q\right)}|\theta_{A}^{\left(q\right)},\theta_{R}^{\left(q\right)},M_{U})},$$

where ${\left\{\left(\theta_{A}^{\left(q\right)}{}^{\prime },\theta_{R}^{\left(q\right)}{}^{\prime },h^{\left(q\right)}{}^{\prime }\right)\right\}}_{q=1}^{k}$ are drawn from the posterior distribution of parameters and latent variables in the model $M_{U}$, i.e., from the distribution given by $p(\theta_{A},\theta_{R},h|y,M_{U})$.

On the other hand, it is easy to show that the Bayes factor in favor of the model $M_{U}$ against the model $M_{R}$ is as follows:

(15) $$\frac{p(y|M_{U})}{p(y|M_{R})}=\int_{\Theta_{A}\times \Theta_{R}\times H}\frac{p(h|\theta_{A},\theta_{R},M_{U})}{p(h|\theta_{R},M_{R})}p\left(\theta_{R},h|y,M_{R}\right)p\left(\theta_{A}|M_{U}\right)\;d\theta_{A}\;d\theta_{R}\;dh,$$

and consequently

(16) $${\hat{BF}}_{U,R,h}=\frac{1}{k}\sum_{q=1}^{k}\frac{p(h^{\left(q\right)}|\theta_{A}^{\left(q\right)},\theta_{R}^{\left(q\right)},M_{U})}{p(h^{\left(q\right)}|\theta_{R}^{\left(q\right)},M_{R})},$$

where ${\left\{\left(\theta_{R}^{\left(q\right)}{}^{\prime },h^{\left(q\right)}{}^{\prime }\right)\right\}}_{q=1}^{k}$ and ${\left\{\theta_{A}^{\left(q\right)}\right\}}_{q=1}^{k}$ are drawn from the posterior distribution $p(\theta_{R},h|y,M_{R})$ and from the prior distribution, $p(\theta {}_{A}|M_{U})$, respectively. However, if the dimension of the vector ${\left(\theta^{\prime },h^{\prime }\right)}^{\prime }$ is high, then the estimators ${\hat{BF}}_{U,R,h}$ and ${\hat{BF}}_{R,U,h}$ tend to suffer from the so-called “simulation pseudo-bias”, similarly to the harmonic mean estimator (see in [26]). This “simulation pseudo-bias” of the estimators will be illustrated on the basis of simulation studies in the next section. To deal with the problem of “pseudo-bias”, we propose drawing from the posterior and prior distributions restricted to a subset of the space of parameters and latent variables with non-zero posterior probability, and correcting the arithmetic mean of the ratio by the posterior probability of the subset visited by the sampler.

Let us assume that $D_{R}\subseteq \Theta_{R}\times H$ and $D_{A}\subseteq \Theta_{A}$, $0<Pr(D_{A}\times D_{R}|M_{U})<\infty$, $0<Pr(D_{A}\times D_{R}|y,M_{U})<\infty$.

Starting from the identity

(17) $$\begin{array}{cc} \frac{p(y|M_{R})}{p(y|M_{U})}\; & \mathrm{Pr}\left(D_{R}|y,M_{R}\right)\mathrm{Pr}\left(D_{A}|M_{U}\right)=\\ \; & =\int_{D_{A}\times D_{R}}\frac{p(y|M_{R})}{p(y|M_{U})}p\left(\theta_{R},h|y,M_{R}\right)p\left(\theta_{A}|M_{U}\right)\;d\theta_{A}\;d\theta_{R}\;dh, \end{array}$$

we obtain

$$\frac{p(y|M_{R})}{p(y|M_{U})}\mathrm{Pr}\left(D_{R}|y,M_{R}\right)\mathrm{Pr}\left(D_{A}|M_{U}\right)=$$

$$=\int_{\Theta_{A}\times \Theta_{R}\times H}I_{D_{A}\times D_{R}}\left(\theta_{A},\theta_{R},h\right)\frac{p\left(\theta_{R},h,y|M_{R}\right)p\left(\theta_{A}|M_{U}\right)}{p\left(y|M_{U}\right)p\left(\theta_{A},\theta_{R},h|y,M_{U}\right)}\;p\left(\theta_{A},\theta_{R},h|y,M_{U}\right)d\theta_{A}\;d\theta_{R}\;dh=$$

$$=\int_{\Theta_{A}\times \Theta_{R}\times H}I_{D_{A}\times D_{R}}\left(\theta_{A},\theta_{R},h\right)\frac{p\left(\theta_{R},h,y|M_{R}\right)p\left(\theta_{A}|M_{U}\right)}{p(\theta_{A},\theta_{R},h,y|M_{U})}\;p\left(\theta_{A},\theta_{R},h|y,M_{U}\right)d\theta_{A}\;d\theta_{R}\;dh.$$

On the basis of the fact that

$$\frac{p\left(\theta_{R},h,y|M_{R}\right)p\left(\theta_{A}|M_{U}\right)}{p(\theta_{A},\theta_{R},h,y|M_{U})}=\frac{p\left(y|\theta_{R},h,M_{R}\right)p\left(h|\theta_{R},M_{R}\right)p\left(\theta_{R}|M_{R}\right)p\left(\theta_{A}|M_{U}\right)}{p\left(y|\theta_{A},\theta_{R},h,M_{U}\right)p\left(h|\theta_{A},\theta_{R},M_{U}\right)p\left(\theta_{A},\theta_{R}|M_{U}\right)},$$

and under assumptions (i)–(vi), we have

$$\frac{p\left(\theta_{R},h,y|M_{R}\right)p\left(\theta_{A}|M_{U}\right)}{p(\theta_{A},\theta_{R},h,y|M_{U})}=\frac{p(h|\theta_{R},M_{R})}{p(h|\theta_{A},\theta_{R},M_{U})}.$$

Therefore,

(18) $$\begin{array}{cc} \frac{p(y|M_{R})}{p(y|M_{U})}=\; & \frac{1}{\mathrm{Pr}\left(D_{R}|y,M_{R}\right)\mathrm{Pr}\left(D_{A}|M_{U}\right)}\times \\ \; & \times \int_{\Theta_{A}\times \Theta_{R}\times H}I_{D_{A}\times D_{R}}\left(\theta_{A},\theta_{R},h\right)\frac{p(h|\theta_{R},M_{R})}{p(h|\theta_{A},\theta_{R},M_{U})}p\left(\theta_{A},\theta_{R},h|y,M_{U}\right)\;d\theta_{A}\;d\theta_{R}\;dh. \end{array}$$

Identity (18) naturally leads to the following estimator of the Bayes factor:

(19) $$\begin{array}{cc} {\hat{BF}}_{R,U,D,h}\; & =\frac{1}{\hat{P}r\left(D_{R}|y,M_{R}\right)\hat{P}r\left(D_{A}|M_{U}\right)}\times \\ \; & \times \sum_{q=1}^{k}\frac{p(h^{\left(q\right)}|\theta_{R}^{\left(q\right)},M_{R})}{p(h^{\left(q\right)}|\theta_{A}^{\left(q\right)},\theta_{R}^{\left(q\right)},M_{U})}I_{D_{A}\times D_{R}}\left(\theta_{A}^{\left(q\right)},\theta_{R}^{\left(q\right)},h^{\left(q\right)}\right), \end{array}$$

where $\left\{\left(\theta_{A}^{\left(q\right)}{}^{\prime }\right),\theta_{R}^{\left(q\right)}{}^{\prime },h^{\left(q\right)}{}^{\prime }\right){\}}_{q=1}^{k}$ are drawn from $p(\theta_{A},\theta_{R},h|y,M_{U})$, or equivalently

(20) $$\begin{array}{cc} {\hat{BF}}_{R,U,D,h}\; & =\frac{\hat{P}r\left(D_{A}\times D_{R}|y,M_{U}\right)}{\hat{P}r\left(D_{R}|y,M_{R}\right)\hat{P}r\left(D_{A}|M_{U}\right)}\times \\ \; & \times \frac{1}{k}\sum_{q=1}^{k}\frac{p(h_{D_{A}\times D_{R}}^{\left(q\right)}|\theta_{R,D_{A}\times D_{R}}^{\left(q\right)},M_{R})}{p(h_{D_{A}\times D_{R}}^{\left(q\right)}|\theta_{A,D_{A}\times D_{R}}^{\left(q\right)},\theta_{R,D_{A}\times D_{R}}^{\left(q\right)},M_{U})}, \end{array}$$

where ${\left\{\left(\theta_{A,D_{A}\times D_{R}}^{\left(q\right)}{}^{\prime },\theta_{R,D_{A}\times D_{R}}^{\left(q\right)}{}^{\prime },h_{D_{A}\times D_{R}}^{\left(q\right)}{}^{\prime }\right)\right\}}_{q=1}^{k}$ are drawn from $p(\theta_{A},\theta_{R},h|y,M_{U})$ truncated to the subset $D_{A}\times D_{R}$. Because the posterior simulation support is the subset $D_{A}\times D_{R}$ of the parameter and latent variable space, most of the ${\left\{\left(\theta_{A,D_{A}\times D_{R}}^{\left(q\right)}{}^{\prime },\theta_{R,D_{A}\times D_{R}}^{\left(q\right)}{}^{\prime },h_{D_{A}\times D_{R}}^{\left(q\right)}{}^{\prime }\right)\right\}}_{q=1}^{k}$ have “similar” values of the ratio of the conditional densities of latent variables, so that, having probabilities $\hat{P}r\left(D_{A}\times D_{R}|y,M_{U}\right)$, $\hat{P}r\left(D_{R}|y,M_{R}\right)$, $\hat{P}r\left(D_{A}|M_{U}\right)$, the simulation process will be numerically more efficient than in the case of unrestricted space of parameters and latent variables. The estimates will not be dominated by few values of the ratio. Therefore, a smaller simulation sample will be required to obtain adequate precision in ${\hat{BF}}_{R,U,D,h}$.

Now suppose that $D\subseteq \Theta_{A}\times \Theta_{R}\times H$ and $0<Pr(D|M_{U})<\infty$, $0<Pr(D|y,M_{U})<\infty$. This time we start with the identity

(21) $$\frac{p(y|M_{U})}{p(y|M_{R})}\mathrm{Pr}\left(D|y,M_{U}\right)=\int_{D}\frac{p(y|M_{U})}{p(y|M_{R})}p\left(\theta_{A},\theta_{R},h|y,M_{U}\right)\;d\theta_{A}\;d\theta_{R}\;dh,$$

and we obtain

$$\frac{p(y|M_{U})}{p(y|M_{R})}\mathrm{Pr}\left(D|y,M_{U}\right)=$$

$$=\int_{\Theta_{A}\times \Theta_{R}\times H}I_{D}\left(\theta_{A},\theta_{R},h\right)\frac{p(\theta_{A},\theta_{R},h,y|M_{U})}{p\left(y|M_{R}\right)p\left(\theta_{R},h|y,M_{R}\right)}\;p\left(\theta_{R},h|y,M_{R}\right)\;d\theta_{A}\;d\theta_{R}\;dh=$$

$$=\int_{\Theta_{A}\times \Theta_{R}\times H}I_{D}\left(\theta_{A},\theta_{R},h\right)\frac{p(\theta_{A},\theta_{R},h,y|M_{U})}{p(\theta_{R},h,y|M_{R})}\;p\left(\theta_{R},h|y,M_{R}\right)d\theta_{A}\;d\theta_{R}\;dh.$$

In this case,

$$\frac{p(\theta_{A},\theta_{R},h,y|M_{U})}{p(\theta_{R},h,y|M_{R})}=\frac{p\left(y|\theta_{A},\theta_{R},h,M_{U}\right)p\left(h|\theta_{A},\theta_{R},M_{U}\right)p\left(\theta_{A},\theta_{R}|M_{U}\right)}{p\left(y|\theta_{R},h,M_{R}\right)p\left(h|\theta_{R},M_{R}\right)p\left(\theta_{R}|M_{R}\right)},$$

and under assumptions (i)–(vi)

$$\frac{p(\theta_{A},\theta_{R},h,y|M_{U})}{p(\theta_{R},h,y|M_{R})}=\frac{p\left(h|\theta_{A},\theta_{R},M_{U}\right)p\left(\theta_{A}|M_{U}\right)}{p(h|\theta_{R},M_{R})}.$$

Thus,

(22) $$\begin{array}{cc} \frac{p(y|M_{U})}{p(y|M_{R})}\; & =\frac{1}{\mathrm{Pr}(D|y,M_{U})}\times \\ \; & \times \int_{\Theta_{A}\times \Theta_{R}\times H}I_{D}\left(\theta_{A},\theta_{R},h\right)\frac{p(h|\theta_{A},\theta_{R},M_{U})}{p(h|\theta_{R},M_{R})}p\left(\theta_{R},h|y,M_{R}\right)p\left(\theta_{A}|M_{U}\right)\;d\theta_{A}\;d\theta_{R}\;dh. \end{array}$$

Given a sample ${\left\{\left(\theta_{R}^{\left(q\right)}{}^{\prime },h^{\left(q\right)}{}^{\prime }\right)\right\}}_{q=1}^{k}$, ${\left\{\theta_{A}^{\left(q\right)}\right\}}_{q=1}^{k}$ from the distributions $p(\theta_{R},h|y,M_{R})$ and $p(\theta {}_{A}|M_{U})$, respectively, then, as results from (22), an estimator of the Bayes factor for the model $M_{U}$ against the model $M_{R}$ can be

(23) $${\hat{BF}}_{U,R,D,h}=\frac{1}{\hat{P}r\left(D|y,M_{U}\right)}\frac{1}{k}\sum_{q=1}^{k}\frac{p(h^{\left(q\right)}|\theta_{A}^{\left(q\right)},\theta_{R}^{\left(q\right)},M_{U})}{p(h^{\left(q\right)}|\theta_{R}^{\left(q\right)},M_{R})}I_{D}\left(\theta_{A}^{\left(q\right)},\theta_{R}^{\left(q\right)},h^{\left(q\right)}\right).$$

Additionally, if $D=D_{A}\times D_{R}\subseteq \Theta_{A}\times \Theta_{R}\times H$, then

(24) $${\hat{BF}}_{U,R,D,h}=\frac{\hat{P}r\left(D_{R}|y,M_{R}\right)\hat{P}r\left(D_{A}|M_{U}\right)}{\hat{P}r\left(D|y,M_{U}\right)}\frac{1}{k}\sum_{q=1}^{k}\frac{p(h_{D}^{\left(q\right)}|\theta_{A,D}^{\left(q\right)},\theta_{R,D}^{\left(q\right)},M_{U})}{p(h_{D}^{\left(q\right)}|\theta_{R,D}^{\left(q\right)},M_{R})},$$

where ${\left\{\left(\theta_{R,D}^{\left(q\right)}{}^{\prime },h_{D}^{\left(q\right)}{}^{\prime }\right)\right\}}_{q=1}^{k}$, ${\left\{\theta_{A,D}^{\left(q\right)}\right\}}_{q=1}^{k}$ are drawn from the distributions determined by $p(\theta_{R},h|y,M_{R})$ and $p(\theta {}_{A}|M_{U})$, respectively, restricted to the subset *D*.

### 2.2. Different Prior Distributions for Common Parameters in Both Models

Now, let us assume that

(v) $p\left(y|\theta_{U},h_{U},M_{U}\right)=p\left(y|\theta_{R},h_{R},M_{R}\right)$, i.e., competing models have the same sampling density and
(vii) $p\left(h|\theta_{A},\theta_{R},M_{U}\right)=p\left(h|\theta_{R},M_{R}\right)$, i.e., in both models, the latent variables have the same prior distribution, instead of (vi) $p\left(\theta_{R}|M_{U}\right)=p\left(\theta_{R}|M_{R}\right)$.

Then, it is easy to show that in the first case

$$\frac{p(y|M_{R})}{p(y|M_{U})}=\frac{1}{\mathrm{Pr}\left(D_{R}|y,M_{R}\right)\;\mathrm{Pr}\left(D_{A}|M_{U}\right)}\times$$

(25) $$\times \int_{\Theta_{A}\times \Theta_{R}\times H}I_{D_{A}\times D_{R}}\left(\theta_{A},\theta_{R},h\right)\frac{p(\theta_{R}|M_{R})}{p(\theta_{R}|M_{U})}p\left(\theta_{A},\theta_{R},h|y,M_{U}\right)\;d\theta_{A}\;d\theta_{R}\;dh,$$

and in the second case

(26) $$\begin{array}{cc} \frac{p(y|M_{U})}{p(y|M_{R})}\; & =\frac{1}{\mathrm{Pr}(D|y,M_{U})}\times \\ \; & \times \int_{\Theta_{A}\times \Theta_{R}\times H}I_{D}\left(\theta_{A},\theta_{R},h\right)\frac{p(\theta_{R}|M_{U})}{p(\theta_{R}|M_{R})}p\left(\theta_{R},h|y,M_{R}\right)p\left(\theta_{A}|M_{U}\right)\;d\theta_{A}\;d\theta_{R}\;dh. \end{array}$$

Basing on identities (25) and (26), the following estimators of the Bayes factors can be formulated:

(27) $$\begin{array}{c} \hat{B}F_{R,U,D,\theta }==\frac{1}{\hat{P}r\left(D_{R}|y,M_{R}\right)\hat{P}r\left(D_{A}|M_{U}\right)}\frac{1}{k}\sum_{q=1}^{k}\frac{p(\theta_{R}^{\left(q\right)}|M_{R})}{p(\theta_{R}^{\left(q\right)}|M_{U})}I_{D_{A}\times D_{R}}\left(\theta_{A}^{\left(q\right)},\theta_{R}^{\left(q\right)},h^{\left(q\right)}\right), \end{array}$$

where ${\left\{\left(\theta_{A}^{\left(q\right)}{}^{\prime },\theta_{R}^{\left(q\right)}{}^{\prime },h^{\left(q\right)}{}^{\prime }\right)\right\}}_{q=1}^{k}$ are drawn from $p(\theta_{A},\theta_{R},h|y,M_{U})$,

(28) $$\hat{B}F_{U,R,D,\theta }=\frac{1}{\hat{P}r\left(D|y,M_{U}\right)}\frac{1}{k}\sum_{q=1}^{k}\frac{p(\theta_{R}^{\left(q\right)}|M_{U})}{p(\theta_{R}^{\left(q\right)}|M_{R})}I_{D}\left(\theta_{A}^{\left(q\right)},\theta_{R}^{\left(q\right)},h^{\left(q\right)}\right),$$

where ${\left\{\left(\theta_{R}^{\left(q\right)}{}^{\prime },h^{\left(q\right)}{}^{\prime }\right)\right\}}_{q=1}^{k}$, ${\left\{\theta_{A}^{\left(q\right)}\right\}}_{q=1}^{k}$ are drawn from $p(\theta_{R},h|y,M_{R})$ and $p(\theta_{A}|M_{U})$, respectively.

### 2.3. Different Conditional Sampling Distributions of the Vector of Observations

Now we assume that conditions (i)–(iv), (vi), and (vii) hold. Thus, the conditional distribution of the observable vector can be different, but the prior distributions for vector of latent variables and common parameters are the same in both competing models. Then, it is easy to show that

(29) $$\begin{array}{cc} \frac{p(y|M_{R})}{p(y|M_{U})}\; & =\frac{1}{\mathrm{Pr}\left(D_{R}|y,M_{R}\right)\;\mathrm{Pr}\left(D_{A}|M_{U}\right)}\times \\ \; & \times \int_{\Theta_{A}\times \Theta_{R}\times H}I_{D_{A}\times D_{R}}\left(\theta_{A},\theta_{R},h\right)\frac{p(y|\theta_{R},h,M_{R})}{p(y|\theta_{A},h,M_{U})}p\left(\theta_{A},\theta_{R},h|y,M_{U}\right)\;d\theta_{A}\;d\theta_{R}\;dh, \end{array}$$

and for the reciprocal of this Bayes factor we have

(30) $$\begin{array}{cc} \frac{p(y|M_{U})}{p(y|M_{R})}\; & =\frac{1}{\mathrm{Pr}(D|y,M_{U})}\times \\ \; & \times \int_{\Theta_{A}\times \Theta_{R}\times H}I_{D}\left(\theta_{A},\theta_{R},h\right)\frac{p(y|\theta_{R},h,M_{U})}{p(y|\theta_{R},h,M_{R})}p\left(\theta_{R},h|y,M_{R}\right)p\left(\theta_{A}|M_{U}\right)\;d\theta_{A}\;d\theta_{R}\;dh. \end{array}$$

Similarly to the above, basing on identities (29) and (30), the following estimators of the Bayes factors can be formulated:

(31) $$\begin{array}{cc} {\hat{BF}}_{R,U,D,y}\; & =\frac{1}{\hat{P}r\left(D_{R}|y,M_{R}\right)\hat{P}r\left(D_{A}|M_{U}\right)}\times \\ \; & \times \frac{1}{k}\sum_{q=1}^{k}\frac{p(y|\theta_{R}^{\left(q\right)},h^{\left(q\right)},M_{R})}{p(y|\theta_{A}^{\left(q\right)},h^{\left(q\right)},M_{U})}I_{D_{A}\times D_{R}}\left(\theta_{A}^{\left(q\right)},\theta_{R}^{\left(q\right)},h^{\left(q\right)}\right), \end{array}$$

where ${\left\{\left(\theta_{A}^{\left(q\right)}{}^{\prime },\theta_{R}^{\left(q\right)}{}^{\prime },h^{\left(q\right)}{}^{\prime }\right)\right\}}_{q=1}^{k}$ are drawn from $p(\theta_{A},\theta_{R},h|y,M_{U})$, and

(32) $${\hat{BF}}_{U,R,D,y}=\frac{1}{\hat{P}r\left(D|y,M_{U}\right)}\frac{1}{k}\sum_{q=1}^{k}\frac{p(y|\theta_{R}^{\left(q\right)},h^{\left(q\right)},M_{U})}{p(y|\theta_{R}^{\left(q\right)},h^{\left(q\right)},M_{R})}I_{D}\left(\theta_{A}^{\left(q\right)},\theta_{R}^{\left(q\right)},h^{\left(q\right)}\right),$$

where ${\left\{\left(\theta_{R}^{\left(q\right)}{}^{\prime },h^{\left(q\right)}{}^{\prime }\right)\right\}}_{q=1}^{k}$, ${\left\{\theta_{A}^{\left(q\right)}\right\}}_{q=1}^{k}$ are drawn from $p(\theta_{R},h|y,M_{R})$ and $p(\theta_{A}|M_{U})$, respectively.

Note that the estimators (19), (22), (27), (28), (31), and (32) are based on the arithmetic mean of the ratio of densities times the indicator function of an arbitrary subset of the space of model parameters and latent variables. Additionally, this arithmetic mean is corrected by the reciprocal of the posterior probability of the subset.

## 3. Simulation Study

In this section, we present a simple simulation study for models with latent variables in which, after having integrated out latent variables analytically, the true values of the marginal likelihoods can easily be assessed using, e.g., the corrected arithmetic mean estimator (CAME [25]). These assessments will be applied to obtain estimates of Bayes factors used as benchmark values for evaluation of the performance of the new method proposed.

Let us consider the Student t model

(33) $$\begin{array}{cc} \; & y_{t}|\mu ,\;v∼iit\left(\mu ,\;1,v\right),\;t=1,2,...,T,\\ \; & \mu ∼N\left(\mu_{0},\;\sigma_{0}^{2}\right),v∼Exp\left(\lambda_{0}\right) \end{array}$$

where t(μ,1,v) denotes the Student t distribution with mode μ, precision 1, and *v* degrees of freedom. $N(\mu_{0},\;\sigma_{0}^{2})$ denotes the Normal distribution with mean $\mu_{0}$ and variance $\sigma_{0}^{2}$, in turn, $Exp(\lambda_{0})$ stands for the Exponential distribution with mean $\lambda_{0}^{-1}$ and variance $\lambda_{0}^{-2}$. The symbol *iid* stands for independent and identically distributed. Thus, the random variables $y_{1},\ldots ,y_{T}$ are independent and have the same Student t distribution.

The Student t distribution can be expressed as a scale mixture of Gaussian distributions by introducing a random variable $h_{t}$ that is inverse-gamma distributed. The model can be written as

(34) $$\begin{array}{cc} \; & y_{t}|\omega_{t},\mu ,\;v∼iiN\left(\mu ,\;h_{t}^{-1}\right),\\ \; & h_{t}|\;v∼iiG\left(v/2,\;v/2\right),\\ \; & \mu ∼N\left(\mu_{0},\sigma_{0}^{2}\right),v∼Exp\left(\lambda_{0}\right), \end{array}$$

where G(v/2,v/2) denotes the gamma distribution with shape v/2 and rate v/2.

To simulate datasets we generated samples of size T=500,1000,2000, data points from model (33) with μ=0,0.25,0.5,v=8, and $\mu_{0}=0;\;\sigma_{0}^{2}=1;\;\lambda_{0}=0.1$.

In turn, to simulate from the posterior distribution the Gibbs algorithm was used and run for 20,000 iterations. The conditional posterior distribution for μ is Normal, i.e.,

(35) $$\mu |h,v,y∼N_{K}\left(\mu_{1},\;\sigma_{1}^{2}\right),$$

where $\sigma_{1}^{2}={\left(\sum_{t=1}^{T}h_{t}+\sigma_{0}^{-2}\right)}^{-1},\;\mu_{1}=\sigma_{1}^{2}\left(\sum_{t=1}^{T}y_{t}h_{t}+\mu_{0}\sigma_{0}^{-2}\right)$, while the conditional posterior distribution for $h_{t}$ is Gamma:

(36) $$h_{t}|\mu ,v,y∼G\left(\left(v+1\right)/2,\;\left[{\left(y_{t}-\mu \right)}^{2}+v\right]/2\right),\;t=1,...,T.$$

An easy computation shows that the conditional posterior distribution of *v* is nonstandard:

(37) $$v|h,\mu ,y∼p\left(v|h,y,\mu \right)\propto {\left(\frac{v}{2}\right)}^{\frac{Tv}{2}}\Gamma {\left(\frac{v}{2}\right)}^{-T}e^{-v\kappa /2},$$

where $\kappa =\sum_{t=1}^{T}h_{t}-\sum_{t=1}^{T}\ln h_{t}+2\lambda_{0}$.

However, reliable numerical methods for generating from this distribution do exist. We use one of them, the rejection sampling proposed by [27].

In the model under consideration, $\theta_{U}={\left(\mu ,v\right)}^{\prime }$, $\theta_{R}=v$, and $h={\left(h_{1},\ldots ,h_{T}\right)}^{\prime }$; thus, the number of latent variables is equal to the number of observations: 500, 1000, and 2000, respectively. The direct computation of the marginal likelihood for model (34) is intensive and unstable due to the presence of latent variables. However, by integrating out the latent variables from the joint distribution of parameters, latent variables, and data, p(y|h,μ,v)p(h,μ,v), the conditional density of the data given parameters only can be written in the following form:

(38) $$p\left(y|\mu ,v\right)=\prod_{t=1}^{T}\frac{\Gamma \left(\frac{v+1}{2}\right)}{\Gamma \left(\frac{v}{2}\right)\sqrt{v\pi }}{\left(1+\frac{{\left(y_{t}-\mu \right)}^{2}}{v}\right)}^{-\frac{v+1}{2}}.$$

Unfortunately, the marginal density of the data (i.e., p(y)) cannot be presented in closed form. However, due to the lack of latent variables as well as thanks to the small number of parameters, the marginal data density value can easily and precisely be evaluated by using the corrected arithmetic mean estimator (CAME [25]). Estimates obtained by the use of the CAME are treated as benchmark values.

In order to show performance of our new estimators of the Bayes factor, we assume that the subset *D* is an intersection of the space of parameters and latent variables, obtained from the condition that the ratio of conditional density functions $p(y|\theta_{U},h,M_{U})$ and $p(y|\theta_{R},h,M_{R})$ is between the lowest and the highest values of the ratio evaluated on the basis of pseudo-random sample ${\left\{\theta^{\left(q\right)},h^{\left(q\right)}\right\}}_{q=1}^{k}$ in both models, and the hyper-cuboid limited by the range of the sampler output, i.e.,

D=(A×B)∩C,

where

$$A=\underset{i}{⊗}\left[\max_{m\in \{M_{U},M_{R}\}}\;\min_{q_{m}}\left\{\theta_{i}^{\left(q_{m}\right)}\right\},\min_{m\in \{M_{U},M_{R}\}}\max_{q_{m}}\left\{\theta_{i}^{\left(q_{m}\right)}\right\}\right],$$

$$B=\underset{t}{⊗}\left[\max_{m\in \{M_{U},M_{R}\}}\;\min_{q_{m}}\left\{h_{t}^{\left(q_{m}\right)}\right\},\min_{m\in \{M_{U},M_{R}\}}\max_{q_{m}}\left\{h_{t}^{\left(q_{m}\right)}\right\}\right],$$

$$C=\{\left(\theta_{U}^{\prime },h^{\prime }\right):L\leq \frac{p(y|\theta_{U},h,M_{U})}{p(y|\theta_{R},h,M_{R})}\leq Q\},$$

$$L=\max_{m\in \{M_{U},M_{R}\}}\min_{q_{m}}\left\{\frac{p(y|\theta_{U}^{\left(q_{m}\right)},h^{\left(q_{m}\right)},M_{U})}{p(y|\theta_{R}^{\left(q_{m}\right)},h^{\left(q_{m}\right)},M_{R})}\right\},$$

$$Q=\min_{m\in \{M_{U},M_{R}\}}\max_{q_{m}}\left\{\frac{p(y|\theta_{U}^{\left(q_{m}\right)},h^{\left(q_{m}\right)},M_{U})}{p(y|\theta_{R}^{\left(q_{m}\right)},h^{\left(q_{m}\right)},M_{R})}\right\}.$$

In the restricted models ($M_{R})$, it is assumed that μ=0, whereas in the unrestricted model ($M_{U}$) μ≠0. The ratio of density functions of the conditional distributions of the observable vector is as follows:

(39) $$\frac{p(y|\theta_{U},h,M_{U})}{p(y|\theta_{R},h,M_{R})}=e^{-\frac{1}{2}\sum_{t=1}^{T}\left[{\left(y_{t}-\mu \right)}^{2}-y_{t}^{2}\right]h_{t}}=e^{-\frac{1}{2}\mu^{2}\sum_{t=1}^{T}h_{t}+\mu \sum_{t=1}^{T}y_{t}h_{t}}.$$

In Table 1, Table 2 and Table 3, we present results obtained by using newly proposed estimators of Bayes factors (i.e., the corrected arithmetic means of the ratio of the densities of the conditional distributions of the observable vector, ${\hat{BF}}_{U,R,D,y}$ and ${\hat{BF}}_{R,U,D,y})$ and uncorrected arithmetic means of the ratios ${\hat{BF}}_{U,R,y}$ and ${\hat{BF}}_{R,U,y}$. Table 1, Table 2 and Table 3 present means, standard deviations, root mean square errors and average errors (relative to the CAM estimates) of the decimal logarithm of estimates obtained in models under consideration. As mentioned earlier, closed-form expressions for the marginal likelihoods do not exist. Therefore, the root mean square errors and average errors are determined relative to the CAM estimates obtained for each marginal likelihood separately.

To estimate $\mathrm{Pr}(D|y,M_{U})$, $\mathrm{Pr}(D_{R}|y,M_{R})$, and $\mathrm{Pr}(D_{A}|M_{U})$, we use simulation from appropriate posterior or prior distributions, e.g.,

(40) $$\hat{P}r\left(D|y,M_{U}\right)=\frac{1}{k}\sum_{q=1}^{k}I_{D}\left(\theta_{A}^{\left(q\right)},\theta_{R}^{\left(q\right)},h^{\left(q\right)}\right),$$

where ${\left\{\left(\theta_{A}^{\left(q\right)}{}^{\prime },\theta_{R}^{\left(q\right)}{}^{\prime },h^{\left(q\right)}{}^{\prime }\right)\right\}}_{q=1}^{k}$ are drawn from the posterior distribution $p(\theta_{A},\theta_{R},h|y,M_{U})$. The remaining probabilities are calculated in a similar manner. Furthermore, we consider uncorrected arithmetic means of the ratios ${\hat{BF}}_{U,R,y}$ and ${\hat{BF}}_{R,U,y}$ to investigate sampling properties of these estimators.

**Figure 1:**
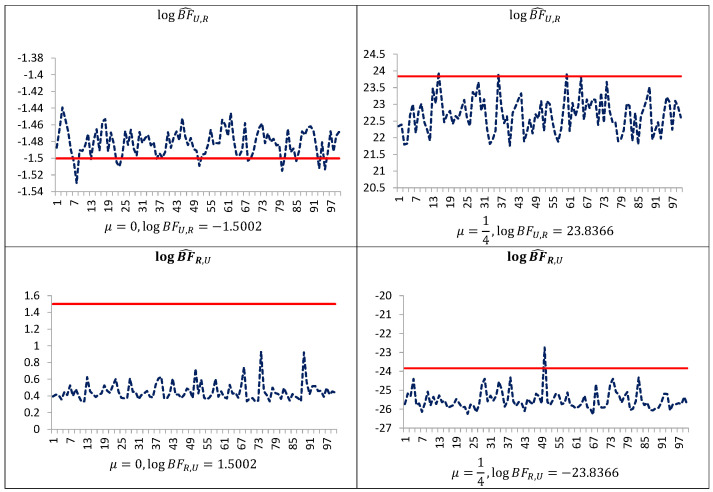
Estimates of the log-Bayes factor in the Student t model, obtained with the use of uncorrected ratios (blue line), and of the corrected arithmetic mean estimator after having integrated out latent variables (red line). Simulation study of the Student t model using 2000 simulated data points (T=2000, which is also equal to the number of latent variables); 20,000 Gibbs sampler iterations for estimation were used. The $logBF_{R,U}$ denotes the decimal logarithm of the Bayes factor in favor of model $M_{R}$ against model $M_{U}$.

**Figure 2:**
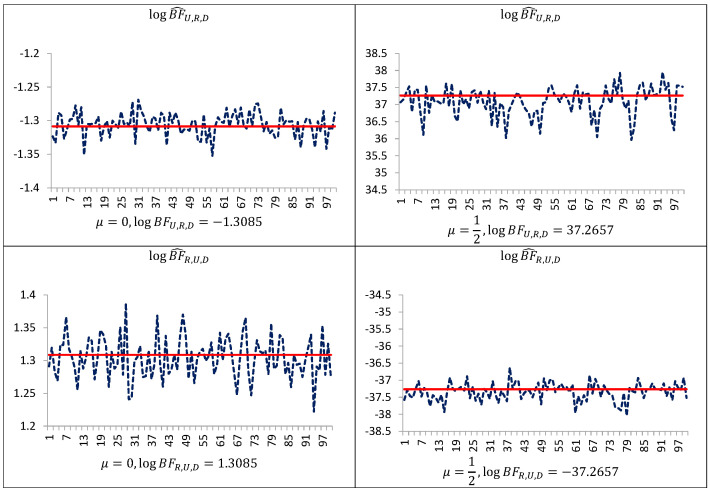
Estimates of the log Bayes factor in the Student t model, obtained with the use of the new estimators (blue line), and of the corrected arithmetic mean estimator after having integrated out latent variables (red line). Simulation study of the Student t model using 1000 simulated data points (T=1000, which is also equal to the number of latent variables); 20,000 Gibbs sampler iterations for estimation were used. The symbol ${\hat{BF}}_{R,U,D}$ denotes corrected estimator of the Bayes factor in favor of model $M_{R}$ against model $M_{U}$. The symbol ${\hat{BF}}_{U,R,D}$ denotes corrected estimator of the Bayes factor in favor of model $M_{U}$ against model $M_{R}$.

**Figure 3:**
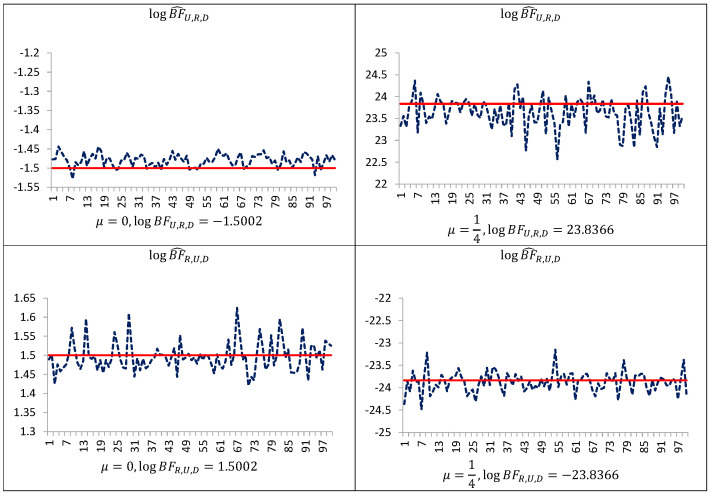
Estimates of the log Bayes factor in the Student t model, obtained with the use of the new estimators (blue line), and of the corrected arithmetic mean estimator after having integrated out latent variables (red line). Simulation study of the Student t model using 1000 simulated data points (T=2000, which is also equal to the number of latent variables); 20,000 Gibbs sampler iterations for estimation were used. The symbol ${\hat{BF}}_{R,U,D}$ denotes corrected estimator of the Bayes factor in favor of model $M_{R}$ against model $M_{U}$. The symbol ${\hat{BF}}_{U,R,D}$ denotes corrected estimator of the Bayes factor in favor of model $M_{U}$ against model $M_{R}$.

Figure 1 indicates that even in such simple models performance of the uncorrected estimators are not satisfactory. The estimator ${\hat{BF}}_{R,U,y}$ is downwardly “pseudo-biased” (respective average errors are negative). On the other hand, our simulation study demonstrates that the performance of the estimators ${\hat{BF}}_{U,R,y}$ and ${\hat{BF}}_{R,U,y}$ can be improved by trimming the posterior simulation support to a given subset of the space of latent variables and parameters, and next making the correction of the arithmetic mean of the ratios using the posterior probabilities of the subset. As can be seen from Table 1, Table 2 and Table 3 and Figure 1, Figure 2 and Figure 3, corrected estimators of the Bayes factors perform very well in comparison to uncorrected ones. The estimators ${\hat{BF}}_{U,R,D,y}$ and ${\hat{BF}}_{R,U,D,y}$ produce estimates which are very close to those obtained on the basis of the CAME, while the estimators ${\hat{BF}}_{U,R,y}$ and ${\hat{BF}}_{R,U,y}$, based on uncorrected arithmetic mean, provide biased estimates.

Finally, note that by using both estimators for the ratio of densities and their reciprocals, i.e., ${\hat{BF}}_{U,R,D,y}$ and ${\hat{BF}}_{R,U,D,y}$, one can check the accuracy of assessments and of adequate (from numerical point of view) selection of the subset *D*. This is because, roughly speaking, the equality $BF_{U,R}=\frac{1}{BF_{R,U}}$ implies that it is natural to use ${\hat{BF}}_{U,R,D,y}$ and $\frac{1}{{\hat{BF}}_{R,U,D,y}}$ to estimate $BF_{U,R}$. Different estimates for $BF_{U,R}$ can indicate numerical inadequacy of selection of the subset *D* and/or too small simulation sample.

## 4. Empirical Illustration: Formal Bayesian Comparison of Hybrid MSV-MGARCH Models

In this section, the new method will be applied in order to formally compare the hybrid Multivariate Stochastic Volatility–Multivariate Generalized Autoregressive Conditional Heteroskedasticity (MSV-MGARCH) models and thus to show that it can be used in practice. The hybrid MSV-MGARCH models were proposed in [28,29,30] for modeling financial time series. These hybrid models are characterized by relatively simple model structures that exploit advantages of both model classes: flexibility of the Multivariate Stochastic Volatility (MSV) class, where volatility is modeled by latent stochastic processes, and relative simplicity of the Multivariate GARCH (MGARCH) class. The simplest specification of MSV-MGARCH model (called LN-MSF-SBEKK) is based on one latent process (Multiplicative Stochastic Factor (MSF)) and on the scalar BEKK [31] covariance structure. The LN-MSF-SBEKK structure is obtained by multiplying the SBEKK conditional covariance matrix $H_{t}$ by a scalar random variable $h_{t}$ such that $\left\{\ln h_{t}\right\}$ is a Gaussian AR(1) latent process with autoregression parameter ϕ. The hybrid LN-MSF-SBEKK specification has been recognized in the literature [32,33,34,35] and proved to be useful in multivariate modeling of financial time series as well as of macroeconomic data [36,37,38,39,40,41]. The drawback of the LN-MSF-MGARCH process is that it cannot be treated as a direct extension of the MGARCH process with the Student t conditional distribution. When ϕ=0, the LN-MSF-SBEKK process reduces itself to the SBEKK process with the conditional distribution being a continuous mixture of multivariate normal distributions with covariance matrices $H_{t}h_{t}$ and $h_{t}$ log-normally distributed. However, the multivariate Student t distribution can be expressed as a scale mixture of normal distributions with the inverted gamma as a mixing distribution. This fact was exploited in [42,43], where the IG-MSF-SBEKK specification was proposed as a natural hybrid extension of the SBEKK process with the Student *t* conditional distribution (t-SBEKK). In the new specification, the latent process $\left\{\ln h_{t}\right\}$ remains an autoregressive process of order one, but it is non-Gaussian. For ϕ=0 the latent variables $h_{t}$ (where t∈Z) are independent and have inverted gamma (IG) distribution. Unfortunately, for ϕ≠0 the unconditional distribution of the latent variables $h_{t}$ is unknown. To summarize, the IG-MSF-SBEKK model is obtained by multiplying the SBEKK conditional covariance matrix $H_{t}$ by a scalar random variable $h_{t}$ coming from such latent process (with autoregression parameter ϕ) that, for ϕ=0, $h_{t}$ has an inverted gamma distribution. Thus, ϕ=0 leads to the t-SBEKK specification, in which the conditional distribution is represented as a continuous mixture of multivariate normal distributions with covariance matrices $H_{t}h_{t}$, where $h_{t}$ is inverted gamma distributed. If ϕ≠0, the latent variables $h_{t}$ (t∈Z) are dependent, so (in comparison to the t-SBEKK model) in the IG-MSF-SBEKK model there exists an additional source of dependence.

Let us consider a two-dimensional autoregressive process $r_{t}=\left(r_{1,t},r_{2,t}\right)$ defined by the equation

(41) $$r_{t}=\delta_{0}+r_{t-1}\Delta +\varepsilon_{t},\;t=1,\cdots ,T,$$

where $\delta_{0}$ and Δ are, respectively, 2×1 and 2×2 matrix parameters, and *T* is the length of the observed time series. The hybrid MSF-MGARCH specification for the disturbance term $\varepsilon_{t}$ is defined by the following equality:

(42) $$\varepsilon_{t}=\zeta_{t}\;H_{t}^{1/2}h_{t}^{1/2},$$

where

(43) $$H_{t}=\left(1-\beta_{1}-\beta_{2}\right)A+\beta_{1}\left(\varepsilon_{t-1}{}^{\prime }\varepsilon_{t-1}\right)+\beta_{2}H_{t-1},$$

(44) $$\ln g_{t}=\phi \ln g_{t-1}+\ln\gamma_{t},$$

$\left\{\zeta_{t}\right\}$ is a Gaussian white noise sequence with mean vector zero and unit covariance matrix; $\left\{\gamma_{t}\right\}$ is a sequence of independent positive random variables; $\gamma_{t}$ is inverted gamma distributed with mean $\frac{v}{v-2}$ for v>2, i.e., $\left\{\gamma_{t}\right\}∼iiIG\left(v/2,\;v/2\right)$, $\zeta_{t}⊥\gamma_{s}$ for all t,s∈{1,⋯,T}, 0<|ϕ|<1; $\beta_{1}$ and $\beta_{2}$ are positive scalar parameters such that $\beta_{1}+\beta_{2}<1$; and *A* is a free symmetric positive definite matrix of order 2. Under (41) and (42), the conditional distribution of $r_{t}$ (given the past of $r_{t}$ and the current latent variable $h_{t})$ is Normal with mean vector $\mu_{t}=\delta_{0}+r_{t-1}\Delta$ and covariance matrix $\Sigma_{t}=H_{t}h_{t}$. For ϕ=0 (this value is excluded in the definition of the hybrid models under consideration) $h_{t}=\gamma_{t}$, so the distribution of $h_{t}$ is an inverted gamma. In this case, $r_{t}$ in (41) is, given its past, an IG mixture of two-variate normal distributions $N(\mu_{t},h_{t}H_{t})$, i.e., it has the two-variate Student t distribution.

The assumptions presented so far determine the conditional distribution of the observations and latent variables given the parameters. Thus, it remains to formulate the prior distributions of parameters. We use the same prior distributions as in [42,43]. Six elements of $\delta =(\delta_{0}vec{\left(\Delta \right)}^{\prime })$ are assumed to be a priori independent of other parameters, with the $N(0,I_{6})$ prior. Matrix *A* has an inverted Wishart prior distribution such that $A^{-1}$ has the Wishart prior distribution with mean $I_{2}$; the elements of $\beta =(\beta_{1},\beta_{2}{)}^{\prime }$ are jointly uniformly distributed over the unit simplex. As regards the initial conditions for $H_{t}$, we take $H_{0}=h_{0}I_{2}$ and treat $h_{0}>0$ as an additional parameter, a priori exponentially distributed with mean 1; ϕ has the uniform distribution over (−1, 1), and for *v* we assume the gamma distribution with mean $\lambda_{a}/\lambda_{v}$, truncated to (2,+∞). We assume that $\lambda_{v}=0.1$ with two cases: $\lambda_{a}=3$ and $\lambda_{a}=1$ (note that $\lambda_{a}=1$ leads to exponential distribution for *v*).

Furthermore, we use the same bivariate data sets as those modeled in [30,42,43]. The first data set consists of the official daily exchange rates of the National Bank of Poland (NBP fixing rates) for the US dollar and German mark in the period 1 February 1996–31 December 2001. The length of the modeled time series of their daily growth rates (logarithmic return rates) is 1482. The second data set consists of the daily quotations of the main index of the Warsaw Stock Exchange (WIG) and the S&P500 index of NYSE—1727 logarithmic returns are modeled from the period 8 January 1999–1 February 2006.

In order to obtain pseudo-random sample from the posterior distribution of parameters and latent variables, we use MCMC simulation techniques described in [42,44] and implemented within the GAUSS programming environment.

Using fully Bayesian methods, we want to answer the question whether the IG-MSF-SBEKK model can be reduced to the t-SBEKK case. Thus, we consider the hypothesis that a scalar parameter ϕ=0 (the t-SBEKK model, $M_{R})$ and the alternative hypothesis that ϕ≠0 (the IG-MSF-SBEKK model, $M_{U})$. For the exchange rate data, the posterior probability that ϕ<0 is approximately 0.001 only and ϕ=0 is included in the highest posterior density (HPD) interval of probability content as high as 0.996. Thus, Lindley’s procedure leads to the conclusion that the t-SBEKK is inadequate. But for the stock data, the posterior probability that ϕ<0 is 0.017 for $\lambda_{a}=3$ and 0.054 for $\lambda_{a}=1$, and ϕ=0 is included in the HPD interval of probability content 0.87 or 0.80, depending on the prior hyperparameter $\lambda_{a}$. In the case of the stock data, Lindley’s testing procedure yields results that the t-SBEKK model cannot be rejected by the data.

Now, our new estimators of the Bayes factors will be used. We start with the assumption that the subset *D* is an intersection of the subspace of parameters and latent variables, obtained by the condition that the ratio of conditional density functions $p(h|\theta_{U},M_{U})$ and $p(h|\theta_{R},M_{R})$ is between the lowest and the highest values of the ratio evaluated at pseudo-random sample ${\left\{\theta^{\left(q\right)},h^{\left(q\right)}\right\}}_{q=1}^{k}$ in both models, and the hyper-cuboid limited by the range of the sampler output, i.e., D=(A×B)∩C, where

$$A=\underset{i}{⊗}\left[\max_{m\in \{M_{U},M_{R}\}}\min_{q_{m}}\left\{\theta_{i}^{\left(q_{m}\right)}\right\},\min_{m\in \{M_{U},M_{R}\}}\max_{q_{m}}\left\{\theta_{i}^{\left(q_{m}\right)}\right\}\right],$$

$$B=\underset{t}{⊗}\left[\max_{m\in \{M_{U},M_{R}\}}\min_{q_{m}}\left\{h_{t}^{\left(q_{m}\right)}\right\},\min_{m\in \{M_{U},M_{R}\}}\max_{q_{m}}\left\{h_{t}^{\left(q_{m}\right)}\right\}\right],$$

$$C=\{\left(\theta_{U}^{\prime },h^{\prime }\right):L\leq \frac{p(h|\theta_{U},M_{U})}{p(h|\theta_{R},M_{R})}\leq Q\},$$

$$L=\max_{m\in \{M_{U},M_{R}\}}\min_{q_{m}}\left\{\frac{p(h^{\left(q_{m}\right)}|\theta_{U}^{\left(q_{m}\right)},M_{U})}{p(h^{\left(q_{m}\right)}|\theta_{R}^{\left(q_{m}\right)},M_{R})}\right\},$$

$$Q=\min_{m\in \{M_{U},M_{R}\}}\max_{q_{m}}\left\{\frac{p(h^{\left(q_{m}\right)}|\theta_{U}^{\left(q_{m}\right)},M_{U})}{p(h^{\left(q_{m}\right)}|\theta_{R}^{\left(q_{m}\right)},M_{R})}\right\}.$$

The ratio of the densities of the conditional distributions of the vector of latent variables is as follows:

(45) $$\frac{p(h|\theta_{U},M_{U})}{p(h|\theta_{R},M_{R})}=\prod_{t=1}^{T}h_{t-1}^{\frac{v}{2}\phi }e^{\frac{v}{2h_{t}}\left(1-h_{t-1}^{\phi }\right)}.$$

In Table 4, we present results obtained by using newly proposed estimators of Bayes factors—the corrected arithmetic means of the ratio of the densities of the conditional distributions of latent variables, $\hat{B}F_{U,R,D}$ and ${\hat{BF}}_{R,U,D}$; the subscript *h* is omitted for convenience. Results obtained on the basis of our new method confirm that in the case of exchange rates, the t-SBEKK model is strongly rejected by the data, whereas it seems that the t-SBEKK specification can be adequate for stock indices. Under equal prior model probabilities, the IG-MSF-SBEKK model for exchange rates is about 14–15 times more probable *a posteriori* than the t-SBEKK model, indicating a strong (but not very strong) evidence against the t-SBEKK model. In turn, for stock indices the decimal logarithm of the Bayes factor of t-SBEKK relative to IG-MSF-SBEKK depends on prior distribution of the number of degrees of freedom, *v*. The Bayes factor in favor of t-SBEKK is equal to about 1.25 and 0.4 in models with $\lambda_{a}=1$ and $\lambda_{a}=3$, respectively. Of course, according to scale of Kass and Raftery (1995) these results indicate evidence for a given model that is negligible: “not worth more than a bare of mention”.

For the sake of comparison, in the last row of the Table 4 the estimates of the Savage–Dickey ratio is presented. The marginal posterior density of ϕ is evaluated at ϕ=0 on the basis of the histogram of the sampled values of ϕ from the posterior distribution. The main conclusions pertaining to the validity of the reduction of the IG-MSF-SBEKK model to the t-SBEKK one are very similar.

Additionally, it is very important to stress that our method makes it possible to compare IG-MSF-SBEKK models whose prior distributions are different (e.g., in respect of the number of degrees of freedom). As can be seen from Table 5, for exchange rates the IG-MSF-SBEKK and t-SBEKK models with exponential distribution for *v* are more probable a posteriori than those with the gamma distribution for *v* with the hyperparameter $\lambda_{a}=3$. In contrary, for stock indices the IG-MSF-SBEKK model with the hyperparameter $\lambda_{a}=3$ is more probable a posteriori than the same model with $\lambda_{a}=1$, but this improvement is negligible.

## 5. Discussion

In this paper, a new method of the estimation of the Bayes factor is proposed. The idea of proposed estimators is based on correction of the arithmetic mean estimator of the ratio of conditional distributions by trimming the posterior sample to a certain subset of the space of parameters and latent variables, *D*, and correcting the arithmetic mean by the posterior probabilities of this subset. The new method makes it possible to compare a finite number of different models with a large number of parameters and/or latent variables in respect to the goodness of fit measured by their posterior probabilities. Our simulation study and empirical illustration show that the method performs well.

In this paper, the question of an adequate selection of the subset *D* used in the method proposed is not addressed. The choice of the subset which minimizes the variance of the estimator remains an open question. The simulation study and empirical example considered in the paper indicate that the choice of *D* as an intersection of the parameter space (with additional restrictions) and the hyper-cuboid limited by the range of the posterior sampler outputs leads to acceptable results.

## Figures and Tables

**Table 1:** Mean (M), standard deviation (SD), average error (AE; corrected arithmetic mean estimator (CAME)—estimated), and root mean square error (RMSE) in the Student t model. The number of observations and of latent variables is equal to T=500.

Estimator and Its Sample Characteristics	for μ=0		for μ=1/2	
	$\log{\mathrm{BF}}_{U,R}$	$\log{\mathrm{BF}}_{R,U}$	$\log{\mathrm{BF}}_{U,R}$	$\log{\mathrm{BF}}_{R,U}$
CAME	−1.256	1.256	21.191	−21.191
$log{\hat{BF}}_{U,R}$: M	−1.254	–	19.826	–
SD	0.012	–	0.748	–
AE	−0.001	–	−1.340	–
RMSE	0.011	–	1.631	–
$log{\hat{BF}}_{U,R,D}$: M	−1.255	–	21.02	–
SD	0.013	–	0.357	–
AE	−0.003	–	−0.209	–
RMSE	0.013	–	0.589	–
$log{\hat{BF}}_{R,U}$: M	–	0.498	–	−22.981
SD	–	0.165	–	0.505
AE	–	−0.776	–	−1.870
RMSE	–	0.783	–	1.935
$log{\hat{BF}}_{R,U,D}$: M	–	1.249	–	−21.238
SD	–	0.032	–	0.284
AE	–	−0.010	–	−0.100
RMSE	–	0.036	–	0.246

Notes: Results obtained for *T* = 500 observations from a Student t distribution with mean *μ*, precision equal to 1 and 8 degrees of freedom. Estimations of the Bayes factors were repeated 100 times. The Bayes factors were estimated with Monte Carlo sampler based on 20,000 iterations. The *log BF_R,U_* denotes the decimal logarithm of the Bayes factor in favor of model *M_R_* against model *M_U_*. The *log BF_U,R_* denotes the decimal logarithm of the Bayes factor in favor of model model *M_U_* against model *M_R_*. ${\hat{BF}}_{U,R}$ denotes the estimator of the Bayes factor calculated using simulation from untruncated distributions, whereas ${\hat{BF}}_{U,R,D}$ denotes the estimator of the Bayes factor based on simulation from truncated distributions to the set *D* (the subscript *y* is omitted for convenience).

**Table 2:** Mean (M), standard deviation (SD), average error (AE; CAME—estimated), and root mean square error (RMSE) in the Student t model. The number of observations and of latent variables is equal to T=1000.

Estimator and Its Sample Characteristics	for μ=0		for μ=1/2	
	$\log{\mathrm{BF}}_{U,R}$	$\log{\mathrm{BF}}_{R,U}$	$\log{\mathrm{BF}}_{U,R}$	$\log{\mathrm{BF}}_{R,U}$
CAME	−1.309	1.309	37.266	−37.266
$log{\hat{BF}}_{U,R}$: M	−1.305	–	34.806	–
SD	0.016	–	0.738	–
AE	0.004	–	−2.485	–
RMSE	0.020	–	2.612	–
$log{\hat{BF}}_{U,R,D}$: M	−1.306	–	37.093	–
SD	0.017	–	0.423	–
AE	0.002	–	−0.151	–
RMSE	0.018	–	0.403	–
$log{\hat{BF}}_{R,U}$: M	–	0.415	–	−40.079
SD	–	0.201	–	0.598
AE	–	−0.813	–	−2.626
RMSE	–	0.850	–	2.753
$log{\hat{BF}}_{R,U,D}$: M	–	1.304	–	−37.341
SD	–	0.032	–	0.258
AE	–	0.0003	–	−0.137
RMSE	–	0.028	–	0.265

Notes: Results obtained for *T* = 1000 observations from a Student t distribution with mean *μ*, precision equal to 1 and 8 degrees of freedom. Meaning of symbols used the same as in Table 1.

**Table 3:** Mean (M), standard deviation (SD), average error (AE; CAME—estimated), and root mean square error (RMSE) in the Student t model. The number of observations and of latent variables is equal to T=2000.

Estimator and Its Sample Characteristics	for μ=0		for μ=1/4	
	$\log{\mathrm{BF}}_{U,R}$	$\log{\mathrm{BF}}_{R,U}$	$\log{\mathrm{BF}}_{U,R}$	$\log{\mathrm{BF}}_{R,U}$
CAME	−1.500	1.500	23.837	−23.837
$log{\hat{BF}}_{U,R}$: M	−1.482	–	22.683	–
SD	0.017	–	0.53	–
AE	0.021	–	−1.204	–
RMSE	0.030	–	1.321	–
$log{\hat{BF}}_{U,R,D}$: M	−1.480	–	23.638	–
SD	0.016	–	0.371	–
AE	0.024	–	−0.134	–
RMSE	0.031	–	0.325	–
$log{\hat{BF}}_{R,U}$: M	–	0.451	–	−25.521
SD	–	0.108	–	0.517
AE	–	−1.070	–	−1.715
RMSE	–	1.072	–	1.755
$log{\hat{BF}}_{R,U,D}$: M	–	1.497	–	−23.874
SD	–	0.039	–	0.226
AE	–	−0.010	–	−0.051
RMSE	–	0.039	–	0.283

Notes: Results obtained for *T* = 2000 observations from a Student t distribution with mean *μ*, precision equal to 1 and 8 degrees of freedom. Meaning of symbols used the same as in Table 1.

**Table 4:** Estimates of Bayes factors for the IG-MSF-SBEKK ($M_{U}$) and t-SBEKK ($M_{R}$) models.

Estimator	Exchange Rates, T=1482		Stock Indices, T=1742	
	$\lambda_{a}=1$	$\lambda_{a}=3$	$\lambda_{a}=1$	$\lambda_{a}=3$
$\log\;{\hat{BF}}_{U,R,D}$	1.147	1.191	−0.110	0.400
(NSE)	(0.017)	(0.024)	(0.012)	(0.014)
$\log\;{\hat{BF}}_{R,U,D}$	−1.134	−1.182	0.092	−0.388
(NSE)	(0.009)	(0.019)	(0.011)	(0.024)
log SD ratio	−1.159	−1.167	0.118	−0.396

Notes: The Bayes factors were estimated with Monte Carlo sampler based on 2,000,000 iterations. The log ${\hat{BF}}_{R,U,D}$ denotes the decimal logarithm of the estimator of the Bayes factor in favor of model *M_R_* against model *M_U_*. NSE denotes the numerical standard error and the log SD ratio stands for the decimal logarithm of the Savage–Dickey density ratio. Scale for the strength of evidence against *M_R_* [45]: 0 < log *BF_U,R_* ≤ 1/2—negligible; 1/2 < log *BF_U,R_* ≤ 1—mild; 1 < log *BF_U,R_* ≤ 2—strong; 2 < log *BF_U,R_*—very strong.

**Table 5:** Estimates of Bayes factors for the IG-MSF-SBEKK models with different prior distributions of the number of degrees of freedom.

	Exchange Rates, T=1482		Stock Indices, T=1742	
Estimator	ϕ≠0	ϕ=0	ϕ≠0	ϕ=0
	(IG-MSF-SBEKK)	(t-SBEKK)	(IG-MSF-SBEKK)	(t-SBEKK)
${\hat{BF}}_{\lambda_{a}=1,\lambda_{a}=3,D}$	8.123	9.517	0.524	1.487
$\log\;{\hat{BF}}_{\lambda_{a}=1,\lambda_{a}=3,D}$	0.910	0.979	−0.281	0.172
${\hat{BF}}_{\lambda_{a}=3,\lambda_{a}=1,D}$	0.123	0.105	1.850	0.622
$\log\;{\hat{BF}}_{\lambda_{a}=3,\lambda_{a}=1,D}$	−0.909	−0.978	0.269	−0.206

Notes: The Bayes factors were estimated with Monte Carlo sampler based on 2,000,000 iterations. The log ${\hat{BF}}_{\lambda_{a}=1,\lambda_{a}=3,D}$ denotes the decimal logarithm of the estimator of the Bayes factor in favor of the IG-MSF-SBEKK model with $\lambda_{a}=1$ against the IG-MSF-SBEKK model with $\lambda_{a}=3$.

## Data Availability

The data are publicly available.
